# Serum 25-hydroxyvitamin D is associated with islet function in postmenopausal women with type 2 diabetes and osteoporosis

**DOI:** 10.3389/fendo.2026.1850506

**Published:** 2026-06-03

**Authors:** Xiuping Yin, Xiao Sun, Fang Gao, Xiaojie Wang, Peipei Chen, Xinxin Yang, Ningning Guo, Guangya Wang, Jinxiu Xu, Dongxia Fu

**Affiliations:** 1 Department of Endocrinology and Diabetes (Second Division), Cangzhou Central Hospital, Cangzhou,Hebei, China; 2 Department of Tuberculosis (First Division), Cangzhou Third Hospital, Cangzhou,Hebei, China; 3 Department of Pediatrics, Cangzhou Heping Hospital, Cangzhou,Hebei, China

**Keywords:** 25-hydroxyvitamin D, osteoporosis, postmenopausal women, type 2 diabetes mellitus, β-cell function

## Abstract

**Background:**

To evaluate the association between serum 25-hydroxyvitamin D [25(OH)D] levels and islet function in postmenopausal women with type 2 diabetes mellitus (T2DM) and osteoporosis.

**Methods:**

A total of 343 consecutively hospitalized postmenopausal women with type 2 diabetes mellitus and osteoporosis between December 2024 and December 2025 were included in the final analysis. Participants were classified into three groups according to serum 25(OH)D levels: sufficiency (>30 ng/mL), insufficiency (20–30 ng/mL), and deficiency (<20 ng/mL). Clinical characteristics, bone mineral density, and islet function-related indices were compared among the groups. Spearman correlation analysis and multivariable linear regression were performed to evaluate the associations between serum 25(OH)D levels and islet function.

**Results:**

No significant differences were found among the three groups in age, duration of T2DM, or glycated hemoglobin (HbA1c) (all P > 0.05), whereas body mass index (BMI) showed a marginal overall difference among the groups (P = 0.047). Lumbar spine, femoral neck, and total hip T-scores were progressively lower across groups with lower serum 25(OH)D levels (all P < 0.001). Fasting insulin (FINS), fasting C-peptide (FCP), homeostasis model assessment of β-cell function (HOMA-β), and homeostasis model assessment of insulin resistance (HOMA-IR) also showed significant intergroup differences (all P < 0.001). Serum 25(OH)D was positively correlated with fasting insulin, fasting C-peptide, and HOMA-β, but negatively correlated with HOMA-IR (all P < 0.001). Multivariable analysis showed that serum 25(OH)D remained associated with islet function and insulin resistance after adjustment for age, BMI, HbA1c, and T2DM duration.

**Conclusion:**

In postmenopausal women with type 2 diabetes mellitus and osteoporosis, lower serum 25(OH)D levels were associated with poorer β-cell function, greater insulin resistance, and lower bone mineral density.

## 1 Introduction

Postmenopausal women represent a high-risk population for impaired bone health because estrogen deficiency disrupts bone remodeling and markedly increases the risk of osteoporosis (1). At the same time, age-related metabolic changes after menopause are associated with an increased risk of glucose metabolism disorders and type 2 diabetes mellitus (T2DM) (2). Osteoporosis and T2DM frequently coexist in postmenopausal women, and their coexistence may further exacerbate metabolic dysfunction and bone fragility, thereby increasing fracture risk and adversely affecting quality of life and long-term prognosis (3, 4).

Serum 25-hydroxyvitamin D [25(OH)D] is a recognized biomarker of vitamin D nutritional status (5) and plays a pivotal role in calcium and phosphate homeostasis, bone turnover, and skeletal remodeling (6). Previous studies have demonstrated that vitamin D deficiency is closely associated with osteoporosis and is linked to reduced bone mineral density, abnormal bone turnover, and an increased risk of fracture (7). Moreover, vitamin D has been implicated in the occurrence and metabolic abnormalities of T2DM (8). Low vitamin D levels may contribute to increased insulin resistance (9), impaired glycemic control, and pancreatic β-cell dysfunction (9, 10).

However, evidence specifically concerning postmenopausal women with coexisting T2DM and osteoporosis remains limited. In this population, estrogen deficiency is common (1, 11), while disturbances in glucose metabolism and bone loss frequently coexist and may interact (3, 12), thereby increasing both metabolic and skeletal risk (13). Therefore, the present study aimed to investigate the association between different serum 25(OH)D levels and indices of islet function in postmenopausal women with T2DM and osteoporosis, thereby providing reference evidence for the comprehensive assessment and clinical management of this high-risk population.

## 2 Materials and methods

### 2.1 Research subjects

This was a single-center retrospective cross-sectional observational study conducted in the Department of Endocrinology, Cangzhou Central Hospital. All consecutively hospitalized postmenopausal women with T2DM and osteoporosis during the study period (December 2024 to December 2025) were screened for eligibility based on predefined inclusion and exclusion criteria, and those who met the criteria were included in the final analysis. A total of 343 patients were included. T2DM was diagnosed according to the criteria of the American Diabetes Association (ADA) (14), including the presence of typical diabetic symptoms (polydipsia, polyuria, polyphagia, and weight loss) with a random blood glucose level ≥11.1 mmol/L, a fasting blood glucose level ≥7.0 mmol/L, or a 2-hour blood glucose level ≥11.1 mmol/L during an oral glucose tolerance test. Osteoporosis was defined as a T-score ≤−2.5, measured by dual-energy X-ray absorptiometry, in accordance with international guidelines (15) and consistent with Chinese guidelines (16). The exclusion criteria were as follows: type 1 diabetes mellitus, gestational diabetes mellitus, or other specific types of diabetes; poor general condition preventing completion of the relevant examinations and assessments; a history of malignant tumors; severe diabetic complications or other diseases that could significantly affect bone metabolism, vitamin D metabolism, or islet function assessment, such as severe diabetic nephropathy, severe neuropathy, recent major cardiovascular or cerebrovascular events, or severe hepatic or renal dysfunction; and use of medications affecting bone metabolism or vitamin D metabolism within the previous 3 months. The study protocol was approved by the Ethics Committee of Cangzhou Central Hospital, Hebei Province (Approval No. 2024-280-02), and conducted in accordance with the Declaration of Helsinki. Written informed consent was obtained in accordance with institutional requirements.

### 2.2 Measurement and grouping of serum 25(OH)D levels

Fasting venous blood samples were collected in the morning during hospitalization. Serum 25-hydroxyvitamin D [25(OH)D] levels were measured using high-performance liquid chromatography–mass spectrometry (HPLC-MS). Participants were categorized into three groups according to serum 25(OH)D levels (16): vitamin D sufficient (>30 ng/mL, n = 99), insufficient (20–30 ng/mL, n = 114), and deficient (<20 ng/mL, n = 130). These thresholds were based on widely accepted clinical criteria (16).

### 2.3 Clinical and biochemical information

Clinical data including age, body mass index (BMI), fasting plasma glucose (FPG), and glycated hemoglobin (HbA1c) were retrieved from the medical records. Bone mineral density (BMD) was measured at the lumbar spine (L1–L4), femoral neck, and total hip using dual-energy X-ray absorptiometry (DXA; Hologic Inc., USA), in accordance with standard operating procedures. The lowest T-score among the measured skeletal sites was used for analysis. T-score was defined as the number of standard deviations (SD) from the mean BMD of a young healthy reference population of the same sex.

### 2.4 Assessment of islet function parameters

During hospitalization, patients who achieved glycemic control [fasting plasma glucose (FPG) < 7.0 mmol/L and 2-hour postprandial plasma glucose (2hPG) < 11.1 mmol/L] underwent a standard 75-g oral glucose tolerance test (OGTT) with simultaneous insulin and C-peptide release tests. Venous blood samples obtained during these assessments were used to measure plasma glucose, insulin, and C-peptide levels. Insulin was measured using a chemiluminescence immunoassay (CLIA), and C-peptide was determined by radioimmunoassay (RIA). The homeostasis model assessment (HOMA) was used to evaluate pancreatic β-cell function and insulin resistance, expressed as HOMA-β and HOMA-IR, respectively. HOMA-β was calculated as 20 × fasting insulin (μU/mL) / [fasting plasma glucose (mmol/L) − 3.5], and HOMA-IR was calculated as fasting plasma glucose (mmol/L) × fasting insulin (μU/mL) / 22.5. Higher HOMA-β values indicate better pancreatic β-cell function, whereas higher HOMA-IR values reflect greater insulin resistance.

### 2.5 Statistical analysis

Statistical analyses were performed using SPSS version 26.0. Continuous variables were tested for normality. Normally distributed variables with homogeneity of variance were expressed as mean ± standard deviation (SD) and compared using one-way analysis of variance (ANOVA), followed by Bonferroni *post hoc* tests. Variables with non-normal distribution or unequal variances were expressed as median (25th–75th percentile) and compared using the Kruskal–Wallis test, followed by Dunn’s *post hoc* test with Bonferroni correction. Spearman correlation analysis was used to assess the relationships between serum 25(OH)D levels and related variables. Multivariable linear regression analyses were performed to evaluate the independent associations between serum 25(OH)D and islet function indicators. Serum 25(OH)D was analyzed as a continuous variable to assess the robustness of the observed associations. Four separate multivariable linear regression models were constructed, with fasting insulin (FINS), fasting C-peptide (FCP), homeostasis model assessment of β-cell function (HOMA-β) and homeostasis model assessment of insulin resistance (HOMA-IR) as dependent variables, respectively, adjusting for age, body mass index, glycated hemoglobin, and duration of type 2 diabetes mellitus. Box plots were used to visually present the distribution and group differences of key variables. A two-sided P < 0.05 was considered statistically significant.

## 3 Results

### 3.1 Participant characteristics

There were no significant differences in age, HbA1c, fasting plasma glucose or duration of T2DM among the three groups (all P > 0.05). BMI showed a marginal overall difference among the three groups in the Kruskal–Wallis test (P = 0.047) (**Table 1**), whereas no significant pairwise differences were observed after Bonferroni correction.

**Table 1:** Comparison of clinical characteristics, bone mineral density, and islet function parameters among different 25(OH)D groups.

Category	Variable	VitD sufficient (n=99)	VitD insufficient (n=114)	VitD deficient (n=130)	F/H value	P value
Clinical	Age(years)	66.00(60.00,70.00)	64.00(60.00,71.50)	67.00(60.00,73.00)	3.78	0.151
	BMI(kg/m²)	24.28(21.88,25.89)	22.89(20.65,24.99)	24.03(21.70,25.39)	6.13	0.047*
	HbA1c (%)	7.30 (6.00, 9.00)	7.30 (5.80, 9.15)	7.36 (6.30, 8.83)	0.88	0.643
	Fasting glucose (mmol/L)	6.50 (5.67, 8.97)	6.13 (5.11, 7.97)	6.25 (5.28, 7.22)	4.67	0.097
	T2DM duration (years)	7.76 ± 2.18	7.74 ± 2.15	7.85 ± 1.94	0.11	0.897
BMD	Lumbar spine T-score	-2.69 (-3.10, -2.30)^a^	-2.99 (-3.37, -2.61)^b^	-3.43 (-3.87, -2.97)^c^	69.99	<0.001***
	Femoral neck T-score	-2.71 (-3.10, -2.45)^a^	-2.93 (-3.36, -2.69)^b^	-3.27 (-3.75, -2.90)^c^	48.25	<0.001***
	Total hip T-score	-2.77 (-3.00, -2.50)^a^	-2.90 (-3.40, -2.70)^b^	-3.16 (-3.56, -2.84)^b^	32.16	<0.001***
Islet function	Fasting insulin	6.64 (5.97, 7.50)^a^	5.37 (4.46, 5.91)^b^	3.89 (3.24, 4.25)^c^	209.05	<0.001***
	HOMA-IR	2.84 ± 0.42^a^	3.46 ± 0.67^b^	4.33 ± 0.61^c^	184.95	<0.001***
	Fasting C-peptide	2.17 ± 0.29^a^	1.81 ± 0.18^b^	1.51 ± 0.18^c^	253.61	<0.001***
	HOMA-β	65.22 (60.11, 69.52)^a^	51.03 (47.15, 55.73)^b^	41.85 (38.42, 45.19)^c^	220.58	<0.001***

Normally distributed variables are presented as mean ± SD; non-normally distributed variables as median (25th–75th percentile). Superscript letters (a, b, c) denote the results of pairwise *post hoc* comparisons within each row: values sharing the same letter are not significantly different from each other, whereas values with different letters are significantly different between the corresponding groups after Bonferroni correction (P < 0.05). BMI, body mass index; HbA1c, glycated hemoglobin; T2DM, type 2 diabetes mellitus; HOMA-IR, homeostasis model assessment of insulin resistance; HOMA-β, homeostasis model assessment of β-cell function; T-score, bone mineral density T-score. *P < 0.05, **P < 0.01, ***P < 0.001.

### 3.2 Comparison of bone mineral density–related parameters among the three groups

Across the three groups with progressively lower serum 25(OH)D levels, lumbar spine, femoral neck, and total hip T-scores showed a stepwise decline. All bone mineral density (BMD) parameters were significantly higher in the vitamin D sufficient group than in both the vitamin D insufficient and vitamin D deficient groups, with the lowest values observed in the vitamin D deficient group. Differences among the three groups were statistically significant for all BMD parameters (all P < 0.001) (**Table 1**).

### 3.3 Comparison of islet function indicators among the three groups

As shown in **Table 1**, significant differences were observed among the three groups in fasting insulin (FINS), fasting C-peptide (FCP), HOMA-β, and HOMA-IR (all P < 0.001). Specifically, FINS, FCP, and HOMA-β decreased progressively across groups with lower serum 25(OH)D levels, whereas HOMA-IR increased significantly across groups with lower serum 25(OH)D levels.

### 3.4 Correlation analysis between 25(OH)D and islet function indicators

**Figure 1:**
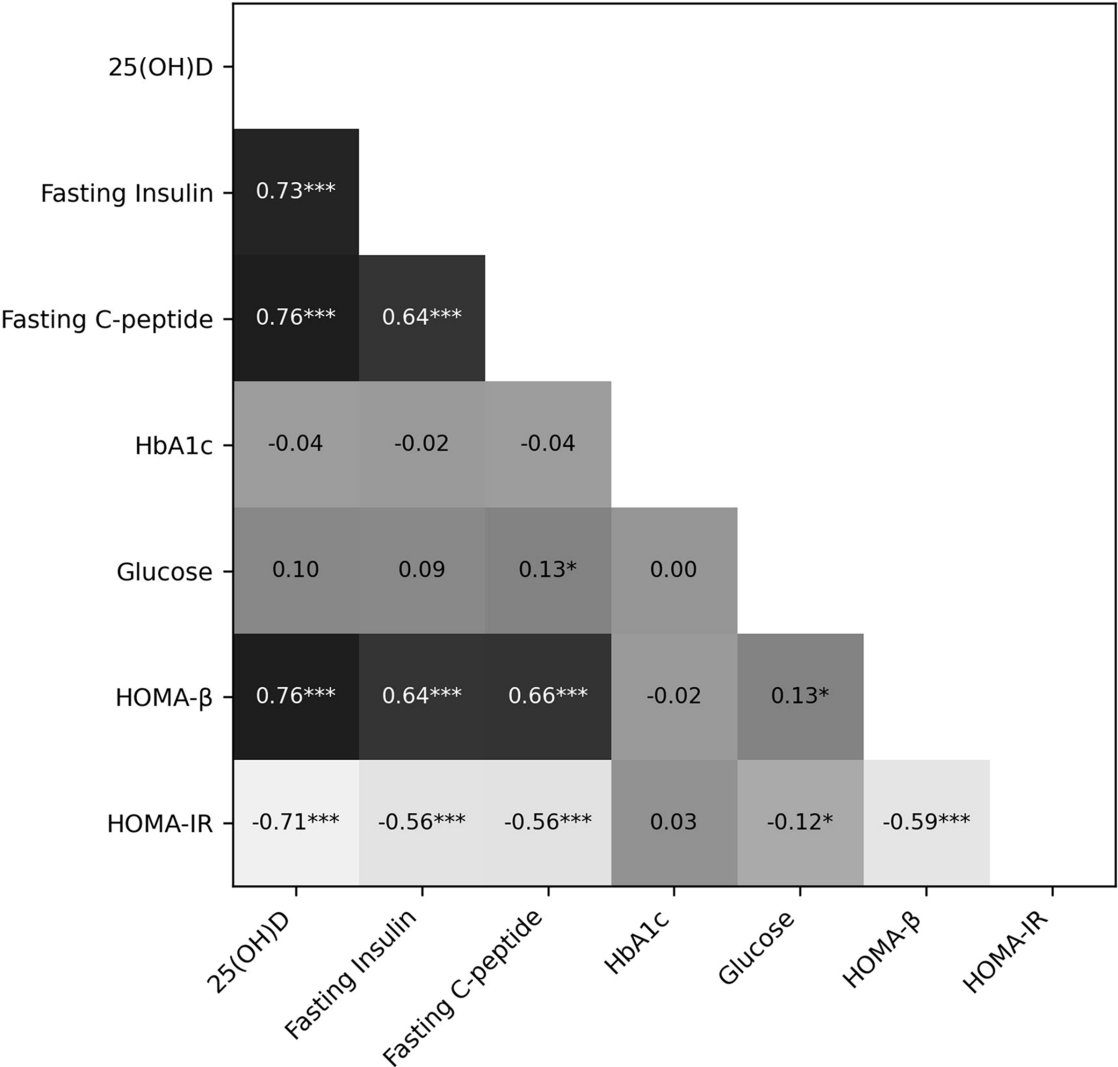
Spearman correlation heatmap between serum 25(OH)D and metabolic indicators. The lower triangle displays Spearman correlation coefficients (r). Asterisks indicate statistical significance (*P<0.05, **P<0.01, ***P<0.001). Shading intensity reflects the strength of the correlations. 25(OH)D, 25-hydroxyvitamin D; HbA1c, glycated hemoglobin; HOMA-β, homeostasis model assessment of β-cell function; HOMA-IR, homeostasis model assessment of insulin resistance.

Spearman correlation analysis revealed that serum 25(OH)D was positively correlated with FINS, FCP, and HOMA-β, and negatively correlated with HOMA-IR (all P < 0.001), as presented in **Table 2** and illustrated in **Figure 1**.

**Table 2:** Spearman correlation between 25(OH)D and metabolic indicators.

Indicator	r (25(OH)D)	P-value
Fasting Insulin	0.73***	<0.001
Fasting C-peptide	0.76***	<0.001
HbA1c	-0.05	0.410
Fasting glucose	0.10	0.060
HOMA-β	0.76***	<0.001
HOMA-IR	-0.71***	<0.001

Spearman correlation coefficients (r) and corresponding P-values are presented. 25(OH)D, 25-hydroxyvitamin D; HbA1c, glycated hemoglobin; HOMA-β, homeostasis model assessment of β-cell function; HOMA-IR, homeostasis model assessment of insulin resistance. *P<0.05, **P<0.01, ***P<0.001.

### 3.5 Multivariable linear regression analysis

**Figure 2:**
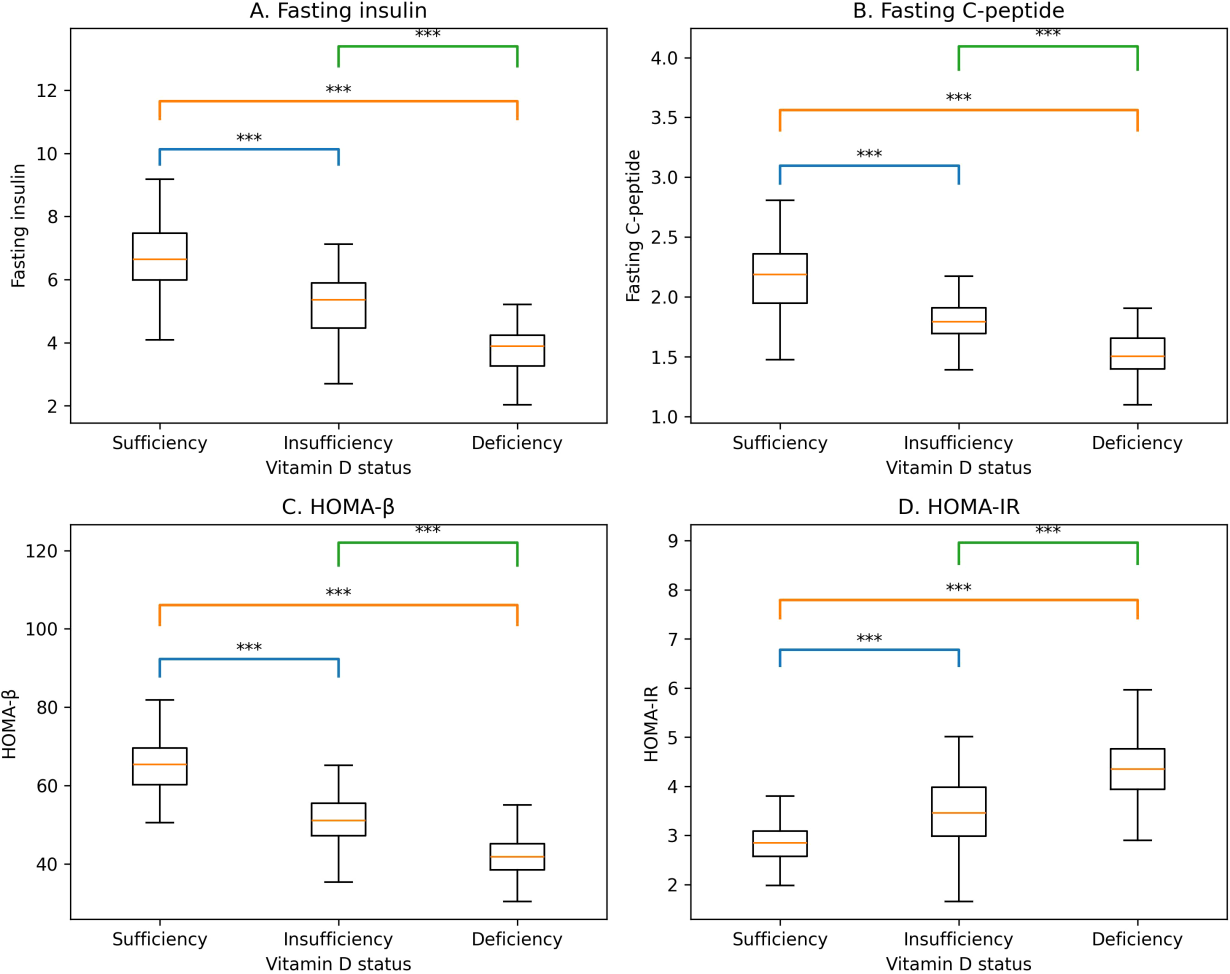
Box plots of islet function indicators across vitamin D status groups. **(A)** Fasting insulin; **(B)** fasting C-peptide; **(C)** HOMA-β; **(D)** HOMA-IR. Differences among groups were assessed using the Kruskal–Wallis test, followed by Dunn’s test with Bonferroni correction. Data are presented as median (IQR). *P<0.05, **P<0.01, ***P<0.001. HOMA-β, homeostasis model assessment of β-cell function; HOMA-IR, homeostasis model assessment of insulin resistance.

In four multivariable linear regression models adjusted for age, BMI, HbA1c, and duration of T2DM, serum 25(OH)D remained positively associated with FINS, FCP, and HOMA-β, and negatively associated with HOMA-IR (all P < 0.001; **Table 3** and **Figure 2**).

**Table 3:** Multivariable linear regression analyses of associations between serum 25(OH)D and islet function indicators.

Variable	B	95% CI	P-value
HOMA-IR	-0.06	(-0.06, -0.05)	<0.001***
HOMA-β	0.84	(0.74, 0.94)	<0.001***
Fasting C-peptide	0.02	(0.02, 0.03)	<0.001***
Fasting Insulin	0.10	(0.09, 0.12)	<0.001***

Fasting insulin (FINS), fasting C-peptide (FCP), homeostasis model assessment of β-cell function (HOMA-β), and homeostasis model assessment of insulin resistance (HOMA-IR) were used as dependent variables in separate multivariable models. Serum 25-hydroxyvitamin D [25(OH)D] was the primary independent variable. The models were adjusted for age, body mass index (BMI), glycated hemoglobin (HbA1c), and duration of type 2 diabetes mellitus. Positive B values indicate positive associations, whereas negative B values indicate inverse associations. *P < 0.05, **P < 0.01, ***P < 0.001.

## 4 Discussion

This study examined the relationship between varying 25(OH)D levels and pancreatic islet function in postmenopausal women with T2DM complicated by osteoporosis. The results demonstrated no statistically significant differences in participant characteristics such as age, HbA1c, and diabetes duration; however, lower 25(OH)D levels were associated with poorer β-cell function and higher insulin resistance. Further, correlation and multiple linear regression analyses indicated an association between 25(OH)D levels and islet function indexes after adjustment for potential confounders, including age, BMI, HbA1c, and diabetes duration. These findings suggest that vitamin D deficiency may be associated not only with lower bone mineral density (BMD), but also with impaired islet function and higher insulin resistance.

Additionally, our study found that the T-scores for the lumbar spine, femoral neck, and total hip bones in the vitamin D-deficient group were lower than those in the vitamin D-sufficient and vitamin D-insufficient groups. This finding indicates that low 25(OH)D status may be associated with poorer bone health in postmenopausal women. Previous studies have shown positive correlations between serum 25(OH)D levels and BMD, with higher 25(OH)D levels associated with increased BMD in the lumbar spine, femoral neck, and total hip. In contrast, vitamin D deficiency has been closely linked to reduced bone mass and elevated osteoporosis risk (17). One possible explanation is that insufficient serum 25(OH)D may lead to elevated parathyroid hormone levels, increased bone resorption, and bone loss, as reported in previous studies (18). Notably, some studies have not observed a significant association between 25(OH)D and BMD, which may be attributable to differences in study populations with respect to age, body composition, and metabolic status (19). In the present study, lower BMD values were observed across multiple skeletal sites in individuals with concurrent abnormalities in glucose and bone metabolism, suggesting that vitamin D deficiency may play a particularly important role in bone impairment in the context of comorbid diabetes and osteoporosis.

Regarding islet function, the vitamin D-sufficient group exhibited the highest fasting C-peptide and HOMA-β levels and the lowest HOMA-IR, whereas the vitamin D-deficient group showed the opposite pattern, and the vitamin D-insufficient group fell between the two. These findings further indicate that vitamin D status is associated with the degree of islet dysfunction. Spearman correlation analysis showed that 25(OH)D levels were positively correlated with β-cell function and negatively correlated with insulin resistance, consistent with previous reports (20–23). A recent meta-analysis reported an association between serum vitamin D levels and insulin resistance in patients with T2DM, suggesting a potential relationship between vitamin D status and insulin sensitivity (22). In postmenopausal women, estrogen deficiency has been associated with impaired glucose and lipid metabolism, and vitamin D insufficiency may coexist with or contribute to a less favorable metabolic profile (23).

Multivariate linear regression analysis further supported these findings. After adjusting for age, diabetes duration, BMI, and HbA1c, serum 25(OH)D levels remained associated with β-cell function, suggesting that this association was not fully explained by these measured metabolic factors. Although BMI showed a marginal overall difference among the vitamin D status groups, pairwise comparisons after Bonferroni correction did not reach statistical significance. The association between 25(OH)D and islet function remained evident after adjustment for BMI, suggesting that this relationship is not solely explained by body weight. Previous studies have shown that obesity may affect vitamin D storage and bioavailability through adipose tissue accumulation (24). Further, elevated BMI is a recognized risk factor for insulin resistance and bone metabolism abnormalities (25–27), suggesting that BMI may serve as a common metabolic background linking these interrelated processes. Nevertheless, as BMI does not fully reflect body composition or visceral adiposity, residual confounding factors related to obesity cannot be entirely excluded.

From a broader pathophysiological perspective, postmenopausal status, glucose metabolism abnormalities, and bone metabolic disturbances are interconnected. Postmenopausal women experience decreased estrogen levels, often accompanied by reduced insulin sensitivity, redistribution of adipose tissue, and chronic low-grade inflammation, which may elevate the risk of metabolic dysregulation. In addition, estrogen deficiency may be associated with altered vitamin D receptor expression (28), reduced intestinal calcium absorption, and impaired bone remodeling balance (29), which may contribute to bone loss. Concurrently, T2DM may diminish bone quality and increase skeletal fragility through persistent hyperglycemia, oxidative stress, and inflammatory responses, suggesting potential reciprocal pathological interactions among these conditions (30–32).

Vitamin D status may be linked to glucose metabolism and bone health through several previously proposed pathways. First, experimental and review studies have suggested that vitamin D may be related to β-cell function and insulin secretion, partly through vitamin D receptor–mediated pathways and calcium homeostasis. Vitamin D status has also been linked to inflammatory and oxidative stress pathways, which are relevant to β-cell dysfunction and insulin resistance (23, 33, 34). Second, vitamin D status has been linked to peripheral insulin sensitivity and insulin resistance, potentially through pathways involving glucose homeostasis in peripheral metabolic tissues (23, 24). Third, vitamin D deficiency may be associated with secondary hyperparathyroidism, increased bone resorption, and bone loss, thereby contributing to impaired skeletal metabolic homeostasis (6, 35).

Additionally, bone metabolism and glucose metabolism are interrelated. Bone-derived factors, particularly osteocalcin, participate in glucose homeostasis and energy regulation, suggesting that bone metabolic abnormalities may further influence islet function through the glucose-bone metabolic network (36, 37). Therefore, in the pathophysiological context of estrogen deficiency, T2DM, and osteoporosis, serum 25(OH)D may serve as a marker reflecting the interaction between glucose metabolism, islet function, and bone health (23, 34, 37). This may, at least partially, provide a possible context for the association observed among 25(OH)D levels, islet function, and bone mass in the present study.

This study may also have clinical implications. Postmenopausal women with T2DM complicated by osteoporosis often present with concurrent bone loss and glucose metabolism abnormalities, with interrelated pathophysiological mechanisms. As a key indicator of vitamin D nutritional status, 25(OH)D levels are associated not only with reduced BMD but also with impaired islet function. Assessment of serum 25(OH)D may therefore provide additional information for integrated metabolic and skeletal risk stratification in this high-risk population. Therefore, comprehensive management of this patient population should include monitoring 25(OH)D levels and identifying vitamin D deficiency alongside standard diabetes and osteoporosis care. In addition, whether correction of vitamin D deficiency improves glucose and bone metabolic outcomes requires further prospective and interventional studies. In particular, patients with low 25(OH)D levels may warrant closer follow-up of glycemic control, BMD, and fracture risk.

This study had several limitations. First, as a single-center, retrospective, cross-sectional study, causal relationships between 25(OH)D levels and islet dysfunction cannot be inferred. In addition, selection bias cannot be excluded, and the generalizability of the findings to community-based or outpatient populations may be limited. Second, although conventional thresholds for vitamin D status were used in this study, the optimal serum 25(OH)D concentration remains debated and may vary according to population characteristics and clinical context (5). Moreover, the 2024 Endocrine Society guideline no longer endorses a fixed 25(OH)D target of 30 ng/mL or specific thresholds for defining vitamin D sufficiency, insufficiency, and deficiency (38). Therefore, the use of traditional cut-off values may have influenced vitamin D classification and the interpretation of between-group differences in islet function and BMD. Third, although analyses adjusted for BMI, residual confounding factors related to unmeasured aspects of adiposity may remain. Moreover, several potentially relevant variables, such as renal function, parathyroid hormone (PTH), serum calcium and phosphate, bone turnover markers, seasonal variation in 25(OH)D levels, sunlight exposure, physical activity, dietary intake, and vitamin D supplementation history, were not available in sufficient detail for inclusion in the regression models. In addition, although T2DM duration and HbA1c did not differ significantly among the vitamin D groups, HbA1c may not fully reflect glycemic variability, and diabetes-related complications may also influence BMD and bone metabolism. Since detailed data on glycemic variability and diabetic complications were not consistently available in this retrospective study, these factors could not be included in the regression models. Therefore, the independence of the association between vitamin D status and BMD from diabetes-related complications should be interpreted with caution. Fourth, subgroup and interaction analyses were not performed. Finally, the specific molecular mechanisms underlying the association of vitamin D status with islet function, bone metabolism, and BMD were not explored. Future multicenter, large-sample, prospective cohort and interventional studies, combined with basic experimental investigations, are required to further elucidate the underlying mechanisms.

## 5 Conclusion

In postmenopausal women with T2DM complicated by osteoporosis, lower serum 25(OH)D levels were associated with stepwise reductions in BMD, impaired β-cell function, and increased insulin resistance. These associations remained significant after adjusting for multiple confounding factors, suggesting that serum 25(OH)D may represent a potential marker of islet function in this patient population. Clinically, assessment and monitoring of vitamin D status may warrant greater attention, and identification and correction of vitamin D deficiency may be considered as part of comprehensive management strategies for metabolic and skeletal health.

## Data Availability

The raw data supporting the conclusions of this article will be made available by the authors, without undue reservation.
