# Supplementary material for: Serum 25-hydroxyvitamin D is associated with islet function in postmenopausal women with type 2 diabetes and osteoporosis
Source: Front Endocrinol (Lausanne). 2026 Jun 3;17:1850506. doi: 10.3389/fendo.2026.1850506 (PMC13272004; doi:10.3389/fendo.2026.1850506)
Supplement: Supplementary file 1 [file Table1.docx]

**Supplementary Material**

# **Supplementary Table 1. Distribution and statistical methods used for each variable**

| **Variable** | **Presentation** | **Overall comparison method** | **Post hoc method** |
| --- | --- | --- | --- |
| Age | Median (25th–75th percentile) | Kruskal–Wallis test | Dunn’s test with Bonferroni correction |
| BMI | Median (25th–75th percentile) | Kruskal–Wallis test | Dunn’s test with Bonferroni correction |
| HbA1c | Median (25th–75th percentile) | Kruskal–Wallis test | Dunn’s test with Bonferroni correction |
| Fasting glucose | Median (25th–75th percentile) | Kruskal–Wallis test | Dunn’s test with Bonferroni correction |
| T2DM duration | Mean ± SD | One-way ANOVA | Bonferroni post hoc test |
| Lumbar spine T-score | Median (25th–75th percentile) | Kruskal–Wallis test | Dunn’s test with Bonferroni correction |
| Femoral neck T-score | Median (25th–75th percentile) | Kruskal–Wallis test | Dunn’s test with Bonferroni correction |
| Total hip T-score | Median (25th–75th percentile) | Kruskal–Wallis test | Dunn’s test with Bonferroni correction |
| Fasting insulin | Median (25th–75th percentile) | Kruskal–Wallis test | Dunn’s test with Bonferroni correction |
| HOMA-IR | Mean ± SD | One-way ANOVA | Bonferroni post hoc test |
| Fasting C-peptide | Mean ± SD | One-way ANOVA | Bonferroni post hoc test |
| HOMA-β | Median (25th–75th percentile) | Kruskal–Wallis test | Dunn’s test with Bonferroni correction |

Note: Normally distributed variables are presented as mean ± SD; non-normally distributed variables are presented as median (25th–75th percentile). BMI, body mass index; HbA1c, glycated hemoglobin; T2DM, type 2 diabetes mellitus; HOMA-IR, homeostasis model assessment of insulin resistance; HOMA-β, homeostasis model assessment of β-cell function; T-score, bone mineral density T-score.

# **Supplementary Table 2. Pairwise post hoc comparisons among vitamin D groups**

| **Variable** | **Comparison** | | **Method** | **Mean difference / test statistic** | **Std. error** | **Adjusted P value** | **Significant** | **95% CI lower** | **95% CI upper** |
| --- | --- | --- | --- | --- | --- | --- | --- | --- | --- |
| BMI | Sufficient vs Insufficient | Dunn's test with Bonferroni correction | | 32.548 | 13.622 | 0.051 | No |  |  |
| BMI | Sufficient vs Deficient | Dunn's test with Bonferroni correction | | 10.235 | 13.227 | 1.000 | No |  |  |
| BMI | Insufficient vs Deficient | Dunn's test with Bonferroni correction | | -22.312 | 12.723 | 0.238 | No |  |  |
| Lumbar spine T-score | Sufficient vs Insufficient | Dunn's test with Bonferroni correction | | 44.495 | 13.622 | 0.003 | Yes |  |  |
| Lumbar spine T-score | Sufficient vs Deficient | Dunn's test with Bonferroni correction | | 108.815 | 13.227 | <0.001 | Yes |  |  |
| Lumbar spine T-score | Insufficient vs Deficient | Dunn's test with Bonferroni correction | | 64.32 | 12.723 | <0.001 | Yes |  |  |
| Femoral neck T-score | Sufficient vs Insufficient | Dunn's test with Bonferroni correction | | 42.995 | 13.622 | 0.005 | Yes |  |  |
| Femoral neck T-score | Sufficient vs Deficient | Dunn's test with Bonferroni correction | | 91.302 | 13.227 | <0.001 | Yes |  |  |
| Femoral neck T-score | Insufficient vs Deficient | Dunn's test with Bonferroni correction | | 48.307 | 12.723 | <0.001 | Yes |  |  |
| Total hip T-score | Sufficient vs Insufficient | Dunn's test with Bonferroni correction | | 47.885 | 13.613 | 0.001 | Yes |  |  |
| Total hip T-score | Sufficient vs Deficient | Dunn's test with Bonferroni correction | | 74.688 | 13.218 | <0.001 | Yes |  |  |
| Total hip T-score | Insufficient vs Deficient | Dunn's test with Bonferroni correction | | 26.803 | 12.715 | 0.105 | No |  |  |
| Fasting insulin | Sufficient vs Insufficient | Dunn's test with Bonferroni correction | | 83.074 | 13.622 | <0.001 | Yes |  |  |
| Fasting insulin | Sufficient vs Deficient | Dunn's test with Bonferroni correction | | 189.144 | 13.227 | <0.001 | Yes |  |  |
| Fasting insulin | Insufficient vs Deficient | | Dunn's test with Bonferroni correction | 106.07 | 12.723 | <0.001 | Yes |  |  |
| HOMA-β | Sufficient vs Insufficient | | Dunn's test with Bonferroni correction | 103.285 | 13.622 | <0.001 | Yes |  |  |
| HOMA-β | Sufficient vs Deficient | | Dunn's test with Bonferroni correction | 196.22 | 13.227 | <0.001 | Yes |  |  |
| HOMA-β | Insufficient vs Deficient | | Dunn's test with Bonferroni correction | 92.935 | 12.723 | <0.001 | Yes |  |  |
| HOMA-IR | Sufficient vs Insufficient | | Bonferroni post hoc test | -0.619 | 0.081 | <0.001 | Yes | -0.814 | -0.425 |
| HOMA-IR | Sufficient vs Deficient | | Bonferroni post hoc test | -1.486 | 0.078 | <0.001 | Yes | -1.675 | -1.297 |
| HOMA-IR | Insufficient vs Deficient | | Bonferroni post hoc test | -0.867 | 0.075 | <0.001 | Yes | -1.048 | -0.685 |
| Fasting C-peptide | Sufficient vs Insufficient | | Bonferroni post hoc test | 0.355 | 0.03 | <0.001 | Yes | 0.283 | 0.428 |
| Fasting C-peptide | Sufficient vs Deficient | | Bonferroni post hoc test | 0.656 | 0.029 | <0.001 | Yes | 0.586 | 0.726 |
| Fasting C-peptide | Insufficient vs Deficient | | Bonferroni post hoc test | 0.301 | 0.028 | <0.001 | Yes | 0.233 | 0.368 |

Note: Vitamin D groups were coded as follows: 1 = sufficient, 2 = insufficient, and 3 = deficient. For variables analyzed using the Kruskal–Wallis test, pairwise comparisons were performed using Dunn’s test with Bonferroni correction. The value in the fourth column is the SPSS pairwise test statistic. For variables analyzed using one-way ANOVA, pairwise comparisons were performed using Bonferroni post hoc tests. The value in the fourth column is the mean difference between the two groups. Adjusted P value < 0.05 was considered statistically significant.

# **Supplementary Table 3. Spearman correlation analysis**

| **Variable** | **Spearman r with 25(OH)D** | **P value** |
| --- | --- | --- |
| Fasting insulin | 0.73 | <0.001 |
| Fasting C-peptide | 0.76 | <0.001 |
| HbA1c | -0.05 | 0.410 |
| Fasting glucose | 0.10 | 0.060 |
| HOMA-β | 0.76 | <0.001 |
| HOMA-IR | -0.71 | <0.001 |

Note: Spearman correlation coefficients (r) and corresponding P values are presented. 25(OH)D, 25-hydroxyvitamin D; HbA1c, glycated hemoglobin; HOMA-β, homeostasis model assessment of β-cell function; HOMA-IR, homeostasis model assessment of insulin resistance. P values <0.001 are shown as <0.001.

# **Supplementary Table 4. Multivariable linear regression analyses of associations between serum 25(OH)D and islet function indicators**

| **Dependent variable** | **Predictor** | **B** | **SE** | **Standardized β** | **t** | **P value** | **95% CI lower** | **95% CI upper** | **Tolerance** | **VIF** |
| --- | --- | --- | --- | --- | --- | --- | --- | --- | --- | --- |
| Fasting insulin | Constant | 2.747 | 0.724 |  | 3.794 | <0.001 | 1.323 | 4.171 |  |  |
| Fasting insulin | 25(OH)D | 0.104 | 0.006 | 0.691 | 17.592 | <0.001 | 0.092 | 0.115 | 0.998 | 1.002 |
| Fasting insulin | Age | -0.007 | 0.007 | -0.039 | -1.003 | 0.316 | -0.021 | 0.007 | 0.997 | 1.003 |
| Fasting insulin | BMI | 0.003 | 0.018 | 0.007 | 0.168 | 0.867 | -0.033 | 0.039 | 0.992 | 1.008 |
| Fasting insulin | HbA1c | 0.041 | 0.03 | 0.054 | 1.354 | 0.177 | -0.019 | 0.101 | 0.986 | 1.014 |
| Fasting insulin | T2DM duration | -0.005 | 0.029 | -0.006 | -0.157 | 0.875 | -0.062 | 0.053 | 0.995 | 1.005 |
| Fasting C-peptide | Constant | 1.648 | 0.161 |  | 10.239 | <0.001 | 1.331 | 1.965 |  |  |
| Fasting C-peptide | 25(OH)D | 0.023 | 0.001 | 0.69 | 17.631 | <0.001 | 0.021 | 0.026 | 0.998 | 1.002 |
| Fasting C-peptide | Age | -0.002 | 0.002 | -0.055 | -1.417 | 0.157 | -0.005 | 0.001 | 0.997 | 1.003 |
| Fasting C-peptide | BMI | -0.01 | 0.004 | -0.093 | -2.369 | 0.018 | -0.018 | -0.002 | 0.992 | 1.008 |
| Fasting C-peptide | HbA1c | 0 | 0.007 | 0.003 | 0.064 | 0.949 | -0.013 | 0.014 | 0.986 | 1.014 |
| Fasting C-peptide | T2DM duration | -0.003 | 0.006 | -0.016 | -0.404 | 0.686 | -0.015 | 0.01 | 0.995 | 1.005 |
| HOMA-β | Constant | 40.834 | 6.162 |  | 6.627 | <0.001 | 28.714 | 52.954 |  |  |
| HOMA-β | 25(OH)D | 0.841 | 0.05 | 0.672 | 16.738 | <0.001 | 0.742 | 0.94 | 0.998 | 1.002 |
| HOMA-β | Age | -0.108 | 0.061 | -0.071 | -1.78 | 0.076 | -0.227 | 0.011 | 0.997 | 1.003 |
| HOMA-β | BMI | -0.039 | 0.157 | -0.01 | -0.246 | 0.806 | -0.347 | 0.27 | 0.992 | 1.008 |
| HOMA-β | HbA1c | -0.07 | 0.258 | -0.011 | -0.272 | 0.786 | -0.579 | 0.438 | 0.986 | 1.014 |
| HOMA-β | T2DM duration | -0.132 | 0.249 | -0.021 | -0.533 | 0.595 | -0.622 | 0.357 | 0.995 | 1.005 |
| HOMA-IR | Constant | 4.438 | 0.402 |  | 11.028 | <0.001 | 3.646 | 5.23 |  |  |
| HOMA-IR | 25(OH)D | -0.056 | 0.003 | -0.68 | -17.123 | <0.001 | -0.063 | -0.05 | 0.998 | 1.002 |
| HOMA-IR | Age | 0.006 | 0.004 | 0.059 | 1.473 | 0.142 | -0.002 | 0.014 | 0.997 | 1.003 |
| HOMA-IR | BMI | -0.003 | 0.01 | -0.013 | -0.332 | 0.740 | -0.024 | 0.017 | 0.992 | 1.008 |
| HOMA-IR | HbA1c | 0.004 | 0.017 | 0.009 | 0.217 | 0.829 | -0.03 | 0.037 | 0.986 | 1.014 |
| HOMA-IR | T2DM duration | 0.021 | 0.016 | 0.052 | 1.305 | 0.193 | -0.011 | 0.053 | 0.995 | 1.005 |

**Overall model significance**

| **Dependent variable** | **Source** | **Sum of squares** | **df** | **Mean square** | **F** | **P value** |
| --- | --- | --- | --- | --- | --- | --- |
| Fasting insulin | Regression | 390.981 | 5 | 78.196 | 62.598 | <0.001 |
| Fasting insulin | Residual | 419.726 | 336 | 1.249 |  |  |
| Fasting insulin | Total | 810.707 | 341 |  |  |  |
| Fasting C-peptide | Regression | 19.694 | 5 | 3.939 | 63.803 | <0.001 |
| Fasting C-peptide | Residual | 20.742 | 336 | 0.062 |  |  |
| Fasting C-peptide | Total | 40.436 | 341 |  |  |  |
| HOMA-β | Regression | 25872.732 | 5 | 5174.546 | 57.201 | <0.001 |
| HOMA-β | Residual | 30395.286 | 336 | 90.462 |  |  |
| HOMA-β | Total | 56268.018 | 341 |  |  |  |
| HOMA-IR | Regression | 115.509 | 5 | 23.102 | 59.865 | <0.001 |
| HOMA-IR | Residual | 129.662 | 336 | 0.386 |  |  |
| HOMA-IR | Total | 245.171 | 341 |  |  |  |

Note: Four separate multivariable linear regression models were constructed, with fasting insulin, fasting C-peptide, HOMA-β, and HOMA-IR as dependent variables. Serum 25(OH)D was the primary independent variable. All models were adjusted for age, body mass index (BMI), glycated hemoglobin (HbA1c), and duration of T2DM. B, unstandardized coefficient; SE, standard error; β, standardized coefficient; CI, confidence interval; VIF, variance inflation factor; HOMA-β, homeostasis model assessment of β-cell function; HOMA-IR, homeostasis model assessment of insulin resistance.
